# Artificial neural network model for predicting changes in ion channel conductance based on cardiac action potential shapes generated via simulation

**DOI:** 10.1038/s41598-021-87578-0

**Published:** 2021-04-09

**Authors:** Da Un Jeong, Ki Moo Lim

**Affiliations:** 1 IT Convergence Engineering, Kumoh National Institute of Technology, Gumi, 39253 Republic of Korea; 2 Medical IT Convergence Engineering, Kumoh National Institute of Technology, Gumi, 39253 Republic of Korea

**Keywords:** Computational models, Machine learning, Biomedical engineering

## Abstract

Many studies have revealed changes in specific protein channels due to physiological causes such as mutation and their effects on action potential duration changes. However, no studies have been conducted to predict the type of protein channel abnormalities that occur through an action potential (AP) shape. Therefore, in this study, we aim to predict the ion channel conductance that is altered from various AP shapes using a machine learning algorithm. We perform electrophysiological simulations using a single-cell model to obtain AP shapes based on variations in the ion channel conductance. In the AP simulation, we increase and decrease the conductance of each ion channel at a constant rate, resulting in 1,980 AP shapes and one standard AP shape without any changes in the ion channel conductance. Subsequently, we calculate the AP difference shapes between them and use them as the input of the machine learning model to predict the changed ion channel conductance. In this study, we demonstrate that the changed ion channel conductance can be predicted with high prediction accuracy, as reflected by an F1 score of 0.985, using only AP shapes and simple machine learning.

## Introduction

The generation of action potentials (APs) in cardiomyocytes comprises five stages, and each stage contains a main ion channel that induces a change in the membrane potential. The membrane potential of the cell maintains a stable negative potential by potassium (K^+^) channels in a normal state (Phase 4). As the membrane potential of the cell reaches the threshold potential of approximately -65 mV, the sodium (Na^+^) channel opens (Phase 0), and the membrane potential changes rapidly to positive as sodium ions enter from the outside of the cell, causing an upstroke (Phase 1). Subsequently, the Na^+^ channel is deactivated and the K^+^ channel is activated. Subsequently, as the calcium (Ca^2+^) channel and delayed rectifier K^+^ outward channel are opened, the AP reaches a plateau by the inward Ca^2+^ current and outward K^+^ current (Phase 2). Thereafter, as the Ca^2+^ channel is deactivated, the membrane potential of the cell, which was in a positive state, returns close to the level of the equilibrium potential of K^+^. Subsequently, the outward K^+^ channel is closed, and the inward K^+^ channel is opened (Phase 3). Finally, it returns to the completely stable state of Phase 4 owing to the inward K^+^ current^1,2^.

APs can be altered by the change in the ion channel conductance due to various causes such as mutations or drugs^3–5^. Advanced studies have been performed that predicted APs based on changes in specific ion channels. In 1994, Luo and Rudy used a mathematical dynamic model to predict changes in ion currents and APs in a ventricular cell based on variations in intracellular Ca^2+^ concentration^6^. Akanda et al. predicted changes in APs due to the biochemical changes caused by toxic drugs using a computational model and suggested that the toxicity or effects of drugs are identifiable by analyzing APs^7^.

There are several studies to predict the ion channel conductance using a machine learning algorithm. Willett and Wilton predicted the five different ion channel targets using two types of activity data through the binary kernel discrimination for chemoinformatics^8^. Redkar et al. predicted the interaction of drug-target such as the enzyme and ion channel using wrapper feature selection, class balancing and compared the performance according to the machine learning algorithms such as Decision Tree, Extreme Gradient Boosting (XGB), Gaussian Naïve Bayes (GNB), K-Nearest Neighbour (KNN), Random Forest (RF) and so on. They suggested the RF algorithm for drug-target interaction prediction^9^. Besides, Khalifa et al. predicted the blocks of sodium ion channel using machine learning-based convolutional quantitative structure–activity relationship model, which has a high accuracy of over 90%^10^. Mei and Zhao successfully predicted the inhibitors of calcium, potassium, and sodium channels by accuracy of 85.7% through the RF and feature extractions based on Chou’s general pseudo amino acid^11^.The aforementioned studies predicted a target ion channel that would be changed by a drug or activity data through the machine learning. However, studies that predict changes in ion channels based on AP shapes have not been conducted. The shape of an AP can vary based on the altered ion channel. In this study, we generated an AP based on the change in ion channel conductance via cell electrophysiological simulation and predicted the ion channel changed by the AP shape using a simple artificial neural network (ANN) model.

## Results

**Figure 1:**
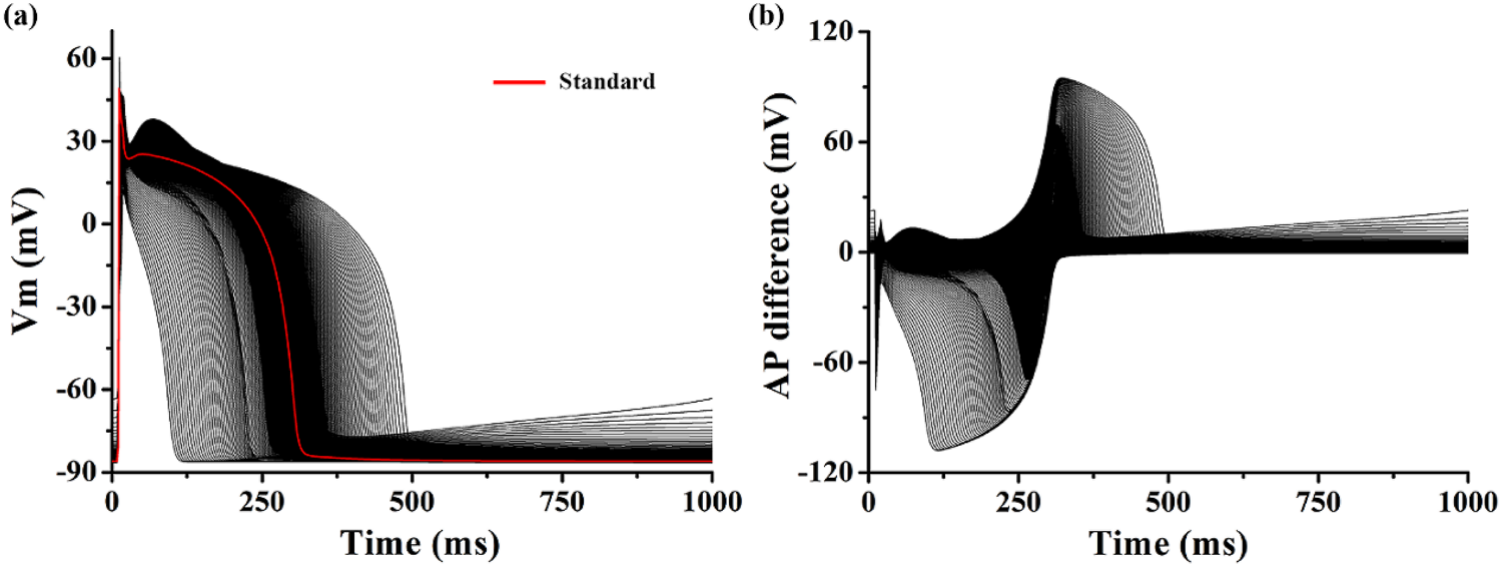
Action potential and action potential difference shapes. Representative action potential shapes (**a**), and representative action potential difference shapes (**b**); action potential difference between standard membrane potential (red line) and changed membrane potential due to change in specific ion conductance.

Using an electrophysiological ventricular cell model, 1980 different APs were obtained based on the change in the conductance of 10 ion channels: G_Ki_, G_Kr_, G_Ks_, G_Na_, G_bNa_, G_CaL_, G_bCa_, G_to_, G_pCa_, and G_pK_ (Table 1). Figure 1a shows the APs during the basic cycle length of 1,000 ms based on the variation in the ion channel electrical conductance. When no change occurred in the ion channel conductance, the action potential duration (APD) was 295 ms (red line in Fig. 1a), and the APD due to the changes in G_Ks_, G_K1_, G_CaL_, G_to_, and G_pK_ were statistically significant from the standard AP (p < 0.05, Table 1). However, the APD under the conditions with changes in G_Ki_, G_Kr_, G_Na_, G_bNa_, G_bCa_, and G_pCa_ had no statistical difference with standard AP (p-values > 0.05). It means it is difficult to classify which ion conductance was changed through only the APD.

**Table 1:** Statistics of APDs based on ion channel conductance.

Abbreviation	Description	Conductance (nS/pF)	APD (ms)		
			Mean	SD	p-value
G_Ks_	Conductance of slow delayed rectifier K^+^ current	0.392	311.78	57.10	5.03E−05
G_Kr_	Conductance of rapid delayed rectifier K^+^ current	0.153	296.90	21.98	0.23
G_K1_	Conductance of maximal inward K^+^ current	5.405	301.84	35.45	0.0074
G_Na_	Conductance of maximal Na^+^ current	14.838	295.13	2.14	0.38
G_bNa_	Conductance of maximal background Na^+^ current	0.00029	295.00	1.15	0.99
G_CaL_	Conductance of maximal L-type Ca^2+^ current	0.0000398	272.36	67.04	4.08E−06
G_bCa_	Conductance of maximal background Ca^2+^ current	0.000592	295.12	5.64	0.76
G_to_	Conductance of transient output K^+^ current	0.073	294.46	1.85	6.34E−05
G_pCa_	Conductance of maximal Ca^2+^ pump current	0.1238	295.07	3.61	0.78
G_pK_	Conductance of maximal K^+^ pump current	0.0146	292.94	2.81	3.97E−20

Figure 1b shows the difference between the standard and simulated APs due to the change in conductance of a specific ion channel. Changes in phases 0 and 1 of the AP due to variations in G_Na_ and G_Ki_ were observed as negative peaks in the AP difference. Besides, changes in phases 0 and 1 due to the variation in G_to_ were observed in the form of positive peaks. The change in the notch of the AP due to the change in G_pK_ was observed in a shape similar to the positive peaks due to the variation in G_to_ in the AP difference; however, relatively low positive peaks were observed again immediately after the occurrence of the peak. The changes in the plateau (phase 2) of the AP due to the variation in G_caL_ was negative in the AP difference, whereas the change in phase 3 of the AP repolarization due to variations in G_Ks_ and G_Kr_ was observed as a positive AP difference. The changes in the resting potential (phase 4) of the AP due to the variation in I_Ki_ was reflected as an increase in the baseline (0 line) of the AP difference.

**Figure 2:**
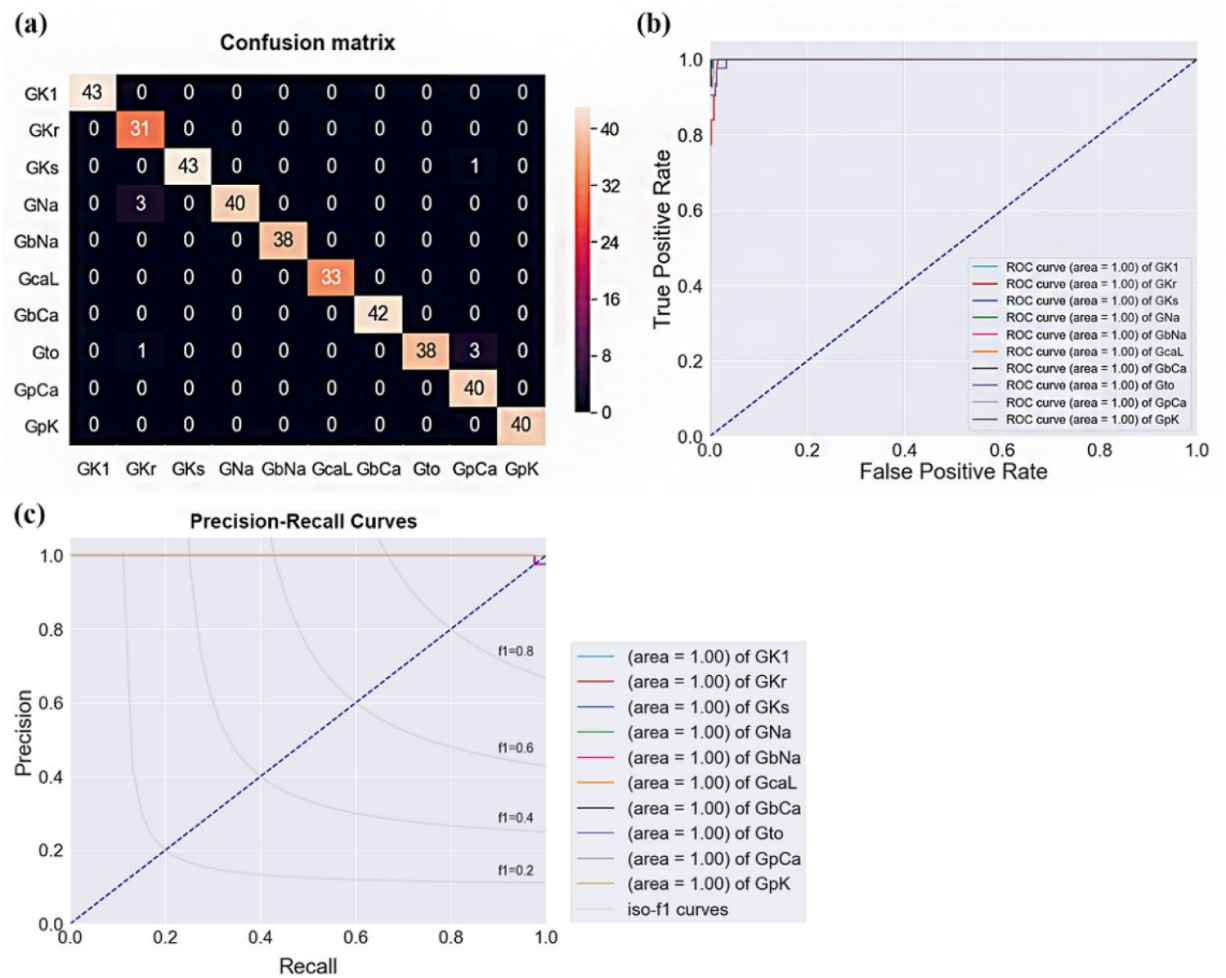
Confusion matrix and receiver operating characteristic (ROC) curves. Confusion matrix (**a**), ROC curves (**b**), and precision-recall curves of the ANN model.

The confusion matrix and receiver operating characteristics (ROC) curve in Fig. 2 show the predicted results of the ion channel, in which the electrical conductance was changed through the ANN model based on the AP difference. Changes in G_Ks_, G_Kr_, G_CaL_, G_to_, G_pCa_, and G_pK_ were accurately classified, and their F1 scores were 1. However, the change in G_bNa_ was the lowest, with an F1 score of 0.930. The predicted F1 scores for G_Na_, G_K1_, and G_bCa_ were 0.981, 0.964, and 0.974, respectively (Fig. 2a). Accordingly, the final F1 score was 0.985 in the prediction of electrical conductance change of the 10 ion channels predicted using our proposed model. Furthermore, the model accuracy was 0.983; the accuracies of each ion channel were 0.99 for G_Kr_, G_Ks_, G_Na_, G_to_, and G_pCa_, and 1.00 for G_K1_, G_bNa_, G_CaL_, G_bCa_, and G_pK_.

The predicted performance of each ion channel is shown in the ROC curve in Fig. 2b. In the ROC curve, y = x lin represents a random classification curve. All 10 classified ROC curves of the ion channel conductance were distributed in the left area of the random classification curve, and all area under the curve was 1, implying that all of the ion channel conductances were accurately classified.

The model was trained with 1,584 randomly extracted data among all the 1,980 data with 10 categories, and the performance of the model was tested using 396 data points. The predictive performance of the model considering the difference in the number of data in the 10 categories was confirmed using a precision-recall curve (Fig. 2c). The predicted precisions of all ion channel conductivities except G_bNa_ were 1, and the precision of G_bNa_ was 0.87. The recall, which is the true positive rate of each ion channel and denotes sensitivity, was 0.93 for G_Ki_, 0.96 for G_Na_, and 0.95 for G_bNCa_. The sensitivity of the other ion channel conductance change predictions was 1. The specificity was 0.98 for G_Ks_, 0.93 for G_Na_, and 0.90 for G_to_, and those for other ion channels’ conductances were 1.

**Figure 3:**
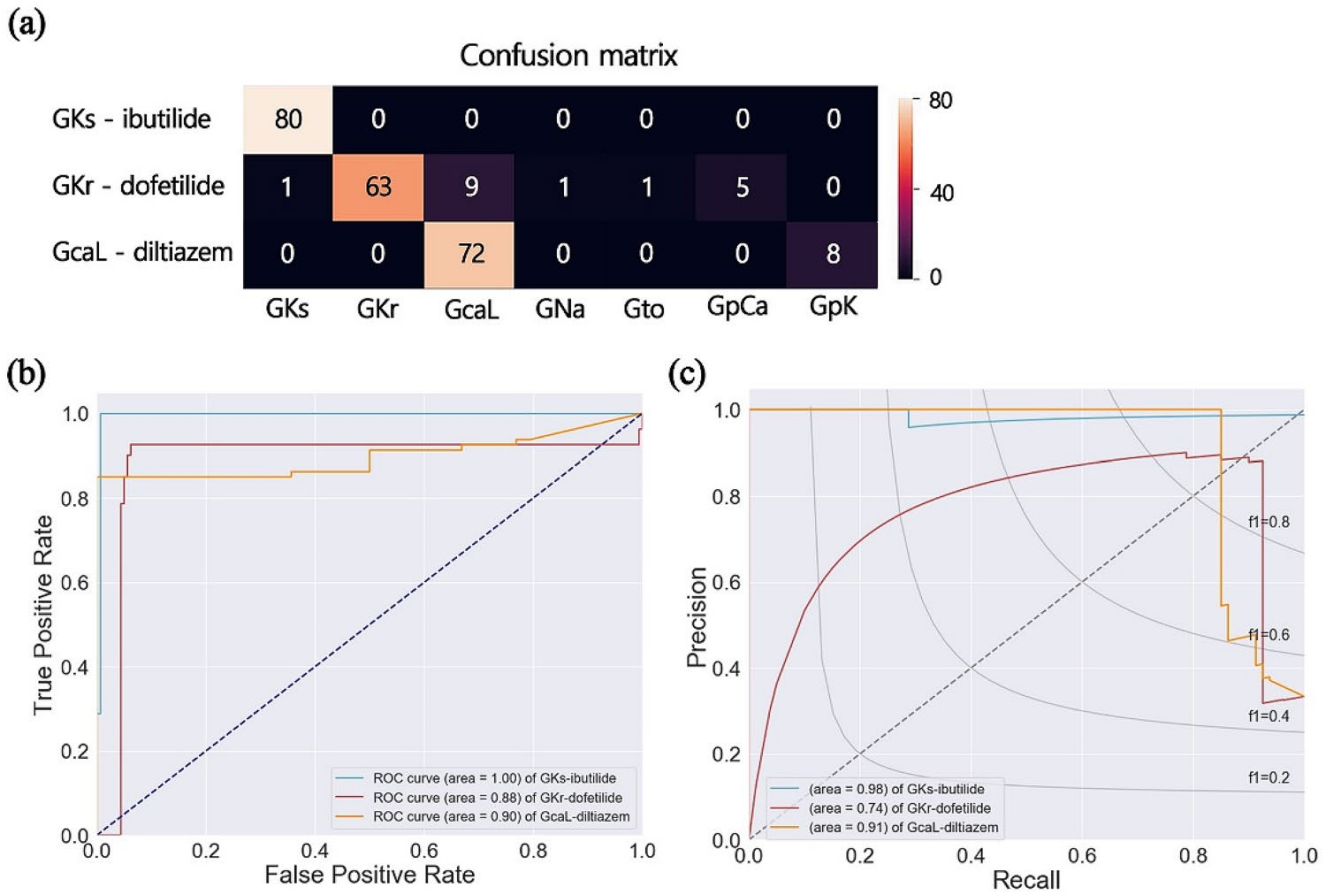
Validation of proposed model using AP shapes with drug effects. Confusion matrix (**a**), ROC curves (**b**), and precision-recall curves (**c**) for predicting the ion channel conductance by drug effects of ibutilide, dofetilide, and diltiazem.

To assess the robustness, we validated the proposed model using APs generated from drug simulations. The APs by drug effects of ibutilide, dofetilide, and diltiazem were simulated using similar protocols suggested by CiPA projects (see the Methods section). Each drug affects the ion channel current; ibutilide inhibits I_Ks_, dofetilide inhibits I_Kr_, and diltiazem inhibits I_CaL_. The classification results for drug effects were shown in Fig. 3. Our proposed model can precisely predict the effect of ibutilide on G_Ks_. Most AP shapes affected by diltiazem were predicted as G_CaL_, but in the case of 8 samples, they were predicted as G_pK_. Most cases of dofetilide were classified to G_Kr_, but other cases of 17 samples were classified as all labels (Fig. 3a). Accordingly, the F1 score was 0.99 for ibutilide, 0.88 for dofetilide, and 0.89 for diltiazem. The accuracies for the prediction of drug effects were 0.99 for ibutilide, and 0.93 for dofetilide and diltiazem.

In the ROC curves, the area under the curve of G_Ks_ (ibutilide) was the highest as 1.00. In G_Ks_ prediction, there was 1 case of false negative and it was reflected in the precision-recall curves (area = 0.98). The area under the ROC curves and precision-recall curves were the lowest in G_CaL_ (diltiazem) as 0.88 and 0.74, respectively. Those for G_Kr_ (dofetilide) were 0.90 for ROC curves and 0.91 for precision-recall curves (Fig. 3b,c). This is because there has never been a case where certain AP shapes were erroneously predicted as G_Kr_, but there were 9 cases when it was erroneously predicted as G_CaL_. Therefore, the specificity was 0.99 for ibutilide, 1.00 for dofetilide, and 0.90 for diltiazem. Furthermore, the sensitivity in predicting the affected G_Ks_ by ibutilide was 1.00, and those in predicting the affected G_Kr_ and G_CaL_ by dofetilide and diltiazem were 0.79 and 0.90, respectively.

## Discussion

In this study, we predicted and classified the changed ion channel’s conductance based on AP shapes using a machine learning algorithm with ANN layers. In this regard, we generated the AP based on changes in 10 ion channels via cell electrophysiology simulation. Subsequently, we predicted the changed ion channel through the AP difference, which was subtracted from the reference AP shape generated under the condition of the cell where no change in the electrical conductance of the ion channel occurred in the AP due to the changed ion channel conductance. The main results of this study are as follows:

1. The AP difference shape, which is the difference between the reference AP and the AP under the changed conditions of the specific ion channel, reflects the change in the AP due to the variation of the ion channel.
2. Using the ANN model based on the AP difference shape, the change in electrical conductance of the ion channel can be classified and predicted accurately by 98%.

Szentandrássy et al. quantitatively confirmed the effect of ionic current based on the short-term variability of the APD through experiments using canine myocytes^12^. They discovered that the short-term variability of the APD was reduced by the negative feedback regulation of I_CaL_, I_Ks_, and I_Kr_, but it was increased by I_Na_ and I_to_. Accordingly, they suggested that these indicators can be used to identify changes in APD caused by drugs. However, it is not trivial to predict the change in electrical conductance of a specific ion channel using the APD only or to statistically confirm the difference (Table 1). When comparing the statistics of the APD based on the change in ion channel conductance through cell simulation, the change in the APD due to G_Kr_, G_Na_, G_bNa_, and G_pCa_ did not differ statistically from the standard AP. Therefore, in this study, ion channels with changed electrical conductance were predicted and classified based on the AP difference, which can reflect the morphological characteristics of the AP shape.The Na^+^ ions are associated with the depolarization upstroke of phase 0 of the AP generation phase^13^. The change in phase 0 based on the variation in G_Na_ appeared as a negative peak of the AP difference. The I_to_ current is known to regulate the phase 1 depolarization of AP generation in cardiomyocytes^14^. Accordingly, the change in I_to_ current due to the variation in G_to_ can affect the depolarization peak of phase 1 in the AP shape, and this change is observed as a positive peak in the AP difference. I_CaL_ is closely associated with the phase 2 plateau of the AP, and it regulates the repolarization duration by controlling the intracellular calcium concentration via the calcium-induced-calcium-released mechanism^15^. The change in I_CaL_ was observed in the form of a negative peak in the AP difference. The K^+^ channels comprised a slow-delayed rectifier channel, a rapid-delayed rectifier channel, and an inward rectifier channel, among which the phase 3 repolarization period of the AP was regulated by the regularization of I_Ks_ and I_Kr_^16,17^. The final phase 4 of the AP generation was regulated by I_Ki_, and the resting potential changed based on the variation in G_Ki_^17^.

The standard AP of the control conditions measured in the experiment can change depending on the environmental conditions of each laboratory or errors such as the handshakes of the experimenter. Because these errors affect the AP shape of other experimental groups under certain conditions, it can affect the analysis and interpretation of the overall experimental results. Therefore, by calculating and using the AP difference between the AP shapes of the experimental and control groups, the effect of correction can be obtained, and the analysis and interpretation accuracies can be improved compared with merely using the AP shape measured in the experiment. Accordingly, the prediction accuracy of the variation in the ion channel conductance can be improved using the AP difference shape instead of using the AP shape.

We tested the ANN model with various hidden layers from one to three. However, even though the number of the hidden layers was increased, the model performance was not significantly improved. Moreover, as the complexity of the model increases, the generalization was rather decreased. Therefore, we proposed the simple ANN model with one hidden layer for predicting the changed ion channel conductance (Supplementary Table S1). Furthermore, we checked the model performance according to the ratio of the training set and testing set which was 60:40, 70:30, 75:25, and 80:20 to check the sensitivity of the proposed model to the training dataset. Then, we concluded there was no significant difference in classification performances according to the ratio of the training set and testing set (Supplementary Table S2).

For robustness, we assessed the proposed model using AP shapes with effects of drugs which are ibutilide, dofetilide, and diltiazem. Our proposed model can predict the inhibited channel current by a certain drug. It is known well for ibutilide to inhibit the slow rectifier potassium current (I_Ks_)^18^, for dofetilide to inhibit the rapid rectifier potassium current (I_Kr_)^19^, and for diltiazem to block the calcium channel (I_CaL_)^20^.

Contraction caused by the electrical stimulation of cardiomyocytes affects cells electrically, a phenomenon known as a mechanoelectrical response. Mechanosensitive ion channels exist in all cells, including cardiomyocytes, and the electrophysiological phenomena of cardiomyocytes can be affected by stretch-activated ion channels^21^. For example, the handling of the intracellular Ca^2+^ can be changed according to the mechano-electric feedback^22^ and the mechano-sensitive ion channels make the depolarization of cardiac fibroblasts by affecting the APD of the myocytes^23^. Furthermore, the mechanosensitivity of the ion channels exist in all cell and affect the single-channel recording technique^21,24^. In this study, this mechanoelectrical response was not considered to predict and classify the variation in ion channel conductance from the difference in AP shape. However, these limitations did not significantly affect the results of the study.

## Methods

### Cellular electrophysiological model

**Figure 4:**
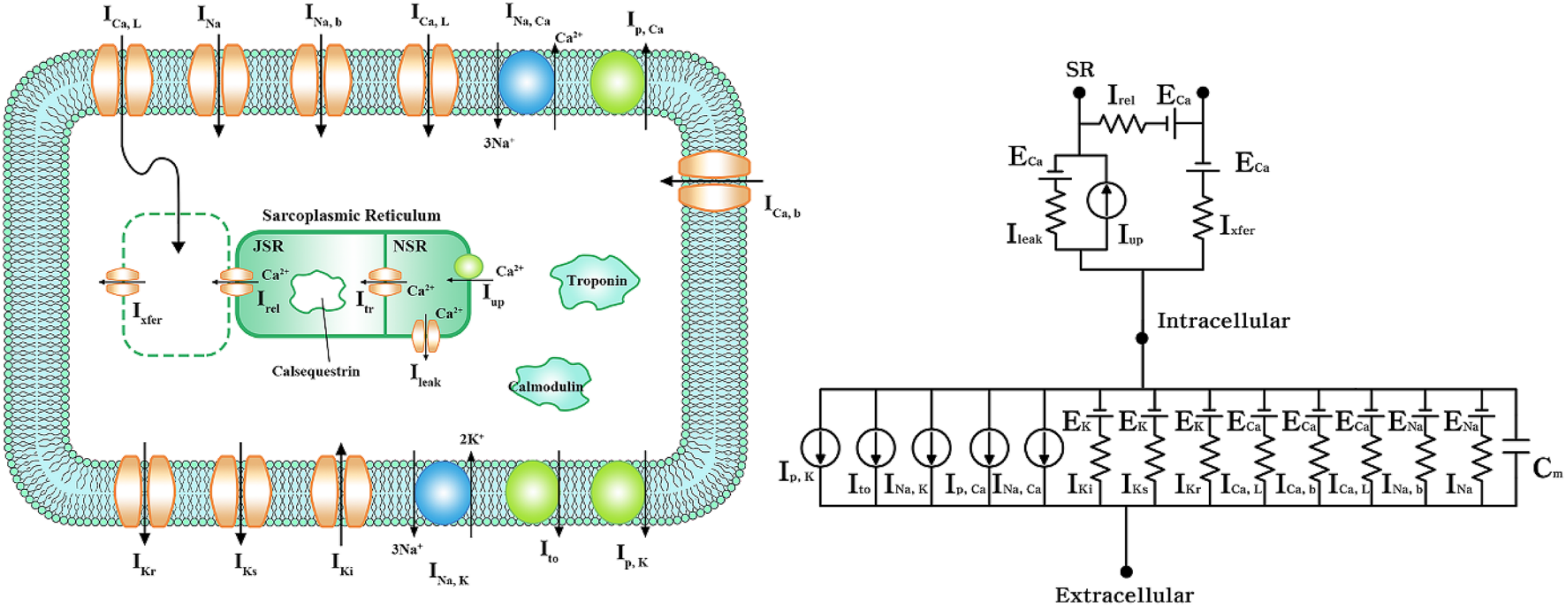
Schematic illustration of the electrophysiological cell model. Electrical schematics representing current, pump, and ion exchanger from Ten Tusscher et al., emulating cell membrane for ion transport and sarcoplasmic reticulum within cardiac cells. “I” represents the ion currents, and “E” the equilibrium potential of each ion; I_Na_, Na^+^ current; I_Ki_, inward rectifier K^+^ current; I_to_, transient outward K^+^ current; I_Kr_, rapid delayed rectifier K^+^ current; I_Ks_, slow delayed rectifier K^+^ current; I_Ca, L_, L-type inward Ca^2+^ current; I_Na,Ca_, Na^+^**–**Ca^2+^ exchange current; I_Na, K_, Na^+^**–**K^+^ exchange current; I_p, K_, K^+^ pump current; I_p, **Ca**_, Ca^2+^ pump current; I_Ca, b_, background Ca^2+^ current; I_Na, b_, background Na^+^ current; E_K_, equilibrium potential of K^+^; E_Ca_, equilibrium potential of Ca^2+^; E_Na_, equilibrium potential of Na^+^; I_leak_, leakage Ca^2+^ current of junctional sarcoplasmic reticulum (JSR); I_rel_, released Ca^2+^ current from JSR; I_up_, absorbed Ca^2+^ current to network sarcoplasmic reticulum (NSR); I_xfer_, diffusible Ca^2+^ current between dyadic subspace and bulk cytoplasm.

Ten Tusscher et al.’s ventricular cell model that mimicked the electrophysiological characteristics of cardiomyocytes was used^25^. The mechanism of ion exchange through the cell membrane was expressed as a lumped-parameter circuit, as shown in Fig. 4, where “I” refers to the ion channel current, and “E” refers to the equilibrium potential of each ion. C_m_ is the membrane capacitance of the cell. The total ion current (I_ion_) through the cell membrane is expressed as follows:

1 $$ I_{ion} = I_{Na} + I_{K1} + I_{to} + I_{Kr} + I_{Ks} + I_{Ca,L} + I_{Na, Ca} + I_{Na,K} + I_{p,Ca} + I_{p,K} + I_{b,Ca} + I_{b,Na} $$

The membrane potential (V_m_) of cardiomyocytes based on the ionic current was calculated using the following equation for electrical conduction^26^:

2 $$ \frac{{dV_{m} }}{dt} = { } - { }\frac{{I_{ion} + I_{stim} }}{{C_{m} }}, $$

where I_stim_ refers to the current caused by an external stimulus.

### Simulation protocol

The ion channel current (I_ion_) based on the electrical conductance (G_ion_) of each ion channel is expressed based on the Hodgkin–Huxley equation as follows^26^:

3 $$ I_{ion} = G_{ion} m_{ion} \left( {V_{m} - E} \right), $$

where M_ion_ is a differential equation expressing the gate state of each ion channel. The reference value of each ion channel conductance was based on that for an endocardial cell suggested by Ten Tusscher et al. (Table 1)^25^. To generate the AP based on the variation in the ion channel conductance, the conductance of each ion channel was decreased by 0.01 from 0.01 to 0.99 times, and increased by 0.01 from 1.01 to 1.99 times, resulting in a total of 1,980 types of AP. The electrophysiology simulations of cardiomyocytes were performed with 10 pacings in a cycle length of 1,000 ms. The simulation results were recorded every 2 ms, and the last 10 pacing cycles were used for prediction. The membrane potential obtained from the cell without any change in the ion channel conductance was defined as the standard AP, and the AP difference was calculated by subtracting the AP owing to the variation in the ion channel conductance from the standard AP. Finally, for the labels for the supervised learning of the ANN model, we put the number from 0 to 9 according to the variation of each ion channel conductance.

To assess the robustness of the proposed model, we performed the electrophysiological simulation under the drug condition and used the generated AP for the validation of the ANN model. To mimic the cardiac cell with drug effects, we used In-vitro experiments data of CiPA (Comprehensive in vitro Proarrhythmia Assay) project group^27^. First, we bootstrapped the half-maximal inhibitory concentration (IC50) and Hill coefficient (H) obtained from In-vitro experiments to IC 50 and H of 2,000 samples. Then, we randomly extracted the IC50 and H of 10 samples among them and used the drug simulation as an input. The drug simulations were conducted under the conditions of 1, 2, 3, 4, 5, 10, 15, and 20 times the Cmax of drugs, which means the highest serum concentration after the drug has been administered and decides as their characteristics values according to the type of drugs. Finally, we obtained 80 AP shapes for each drug condition and the total AP shapes with drug effects were 240. The parameters for drug simulation were set the same as the original electrophysiological simulation we mentioned in the previous paragraph. The answer labels were put according to inhibited channel currents by each drug; G_Ks_ for ibutilide^18^, G_Kr_ for dofetilide^19^, and G_CaL_ for diltiazem^20^.

### ANN model construction

The ANN model for predicting the change in the ion channel conductance comprised one fully connected layer with 130 neurons, and the ReLU activation function^28^ was used in the hidden layer. The classification results were generated through the output layer, which was composed of 10 neurons, where the activation function was used with the Softmax function. The categorical cross-entropy function was used as the loss function, and Adam^29^ with a learning rate of 0.001 was used as the optimization function. Seventy percent of the total data was used for model training and 20% for model testing. To prevent overfitting due to the limited amount of data, the performance of the model was evaluated using tenfold cross-validation.

The classification performance using the proposed ANN model was evaluated based on indices of accuracy, precision, recall, and F1 score to prevent the model from being overestimated owing to the imbalanced data in each label^30^.

4 $$ Recall \left( {sensitivity} \right) = \frac{1}{n}\mathop \sum \limits_{i = 1}^{n} \frac{{\left| {Y_{i} \cap h\left( {x_{i} } \right)} \right|}}{{\left| {Y_{i} } \right|}} $$

5 $$ Precision = \frac{1}{n}\mathop \sum \limits_{i = 1}^{n} \frac{{\left| {Y_{i} \cap h\left( {x_{i} } \right)} \right|}}{{\left| {h\left( {x_{i} } \right)} \right|}} $$

6 $$ F1 score = \left( {\frac{{Recall^{ - 1} + Precision^{ - 1} }}{2}} \right)^{ - 1} = 2 \cdot \frac{Recall \cdot Precision}{{Recall + Precision}} $$

In Eqs. (4) and (5), *Y*_*i*_ is the true label, and *h(x*_*i*_*)* is the value predicted through the model. The correct answer predicted through the model can be expressed as $Y_{i} \cap h\left( {x_{i} } \right)$. Accordingly, the overall classification performance for the ion channel conductance was calculated as follows:

7 $$ F1_{total} = \frac{{F1_{GKs} + F1_{GKr} + F1_{GK1} + F1_{GNa} + F1_{bNa} + F1_{CaL} + F1_{bCa} + F1_{Gto} + F1_{GpCa} + F1_{GpK} }}{10} $$

## Supplementary Information

Supplementary Information

## Data Availability

All datasets used in the study were generated through simulations performed by the authors based on the methods described in the text.

## References

[1] Shih H-T (1994). Anatomy of the action potential in the heart. Texas Heart Inst. J..

[2] Jose, J., Mario, D., Justus, A., Omer, B. & Kalifa, J. *Basic cardiac electrophysiology for the clinician*. *Cardiovascular Medicine* (2009).

[3] Atrial G (1985). Configurations of single. Cell.

[4] Hong K (2005). De novo KCNQ1 mutation responsible for atrial fibrillation and short QT syndrome in utero. Cardiovasc. Res..

[5] Hasegawa K (2014). A novel KCNQ1 missense mutation identified in a patient with juvenile-onset atrial fibrillation causes constitutively open I Ks channels. Heart Rhythm.

[6] Luo CH, Rudy Y (1994). A dynamic model of the cardiac ventricular action potential: I. Simulations of ionic currents and concentration changes. Circ. Res..

[7] Akanda N, Molnar P, Stancescu M, Hickman JJ (2009). Analysis of toxin-induced changes in action potential shape for drug development. J. Biomol. Screen..

[8] Willett P (2007). Prediction of ion channel activity using binary kernel discrimination. J. Chem. Inf. Model..

[9] Redkar S, Mondal S, Joseph A, Hareesha KS (2020). A machine learning approach for drug-target interaction prediction using wrapper feature selection and class balancing. Mol. Inf..

[10] Khalifa N, Kumar Konda LS, Kristam R (2020). Machine learning-based QSAR models to predict sodium ion channel (Nav1.5) blockers. Future Med. Chem..

[11] Mei J, Fu Y, Zhao J (2018). Analysis and prediction of ion channel inhibitors by using feature selection and Chou’s general pseudo amino acid composition. J. Theor. Biol..

[12] Szentandrássy N (2015). Contribution of ion currents to beat-to-beat variability of action potential duration in canine ventricular myocytes. Pflugers Arch. Eur. J. Physiol..

[13] Liu YM, DeFelice LJ, Mazzanti M (1992). Na channels that remain open throughout the cardiac action potential plateau. Biophys. J..

[14] Sah R (2003). Regulation of cardiac excitation-contraction coupling by action potential repolarization: role of the transient outward potassium current (Ito). J. Physiol..

[15] Trautwein W, Hescheler J (1990). Regulation of cardiac L-type calcium current by phosphorylation and G proteins. Annu. Rev. Physiol..

[16] Grunnet M (2010). Repolarization of the cardiac action potential. Dose an increase in repolarization capacity constitute a new anti-arrhythmic principle?. Acta Physiol..

[17] Surawicz B (1992). Role of potassium channels in cycle length dependent regulation of action potential duration in mammalian cardiac purkinje and ventricular muscle fiber. Cardiovasc. Res..

[18] Murray KT (1998). Ibutilide. Circulation.

[19] Roukoz H, Saliba W (2007). Dofetilide: a new class III antiarrhythmic agent. Expert Rev. Cardiovasc. Ther..

[20] Basile J (2004). The role of existing and newer calcium channel blockers in the treatment of hypertension. J. Clin. Hypertens. (Greenwich).

[21] Sachs F (2010). Stretch-activated ion channels: what are they?. Physiology.

[22] Xie LH, Sato D, Garfinkel A, Qu Z, Weiss JN (2008). Intracellular Ca alternans: coordinated regulation by sarcoplasmic reticulum release, uptake, and leak. Biophys. J..

[23] Miragoli M, Gaudesius G, Rohr S (2006). Electrotonic modulation of cardiac impulse conduction by myofibroblasts. Circ. Res..

[24] Morris CE, Horn R (1991). Failure to elicit neuronal macroscopic mechanosensitive currents anticipated by single-channel studies. Science (80-).

[25] Ten Tusscher KHWJ (2004). A model for human ventricular tissue. AJP Heart Circ. Physiol..

[26] Hodgkin AL, Huxley AF (1952). A quantitative description of membrane current and its application to conduction and excitation in nerve. J. Physiol..

[27] Li Z (2019). Assessment of an in silico mechanistic model for proarrhythmia risk prediction under the CiPA initiative. Clin. Pharmacol. Ther..

[28] Xavier G, Antoine B, Yoshua B (2011). Deep sparse rectifier neural networks. Int. Conf. Artif. Intell. Stat. AISTATS.

[29] Kingma, D. P. & Ba, J. L. Adam: A method for stochastic optimization. in *3rd International Conference on Learning Representations, ICLR 2015 - Conference Track Proceedings* 1–15 (2015).

[30] Fawcett T (2006). An introduction to ROC analysis. Pattern Recognit. Lett..

