# Supplementary material for: Artificial neural network model for predicting changes in ion channel conductance based on cardiac action potential shapes generated via simulation
Source: Sci Rep. 2021 Apr 9;11:7831. doi: 10.1038/s41598-021-87578-0 (PMC8035260; doi:10.1038/s41598-021-87578-0)
Supplement: Supplementary file 1 — Supplementary Information [file 41598_2021_87578_MOESM1_ESM.pdf]

Artificial Neural Network Model for Predicting Changes in Ion channel Conductance Based on Cardiac Action Potential Shapes Generated via Simulation

Da Un Jeong[1], Ki Moo Lim[2*]

[1]Kumoh National Institute of Technology, IT convergence engineering, Gumi, 39253, Republic of Korea.
[2]Kumog National Institute of Technology, Medical IT convergence engineering, Gumi, 39253, Republic of Korea.
[*]kmlim@kumoh.ac.kr

Supplementary Tables

Supplementary Table S1. Sensitivity to the structures of ANN model

	Number of hidden layers	F1 score	Accuracy
Case 1	1	0.985	0.983
Case 2	2	0.982	0.983
Case 3	3	0.967	0.968
Case 4	4	0.968	0.968

Supplementary Table S2. Sensitivity to the ratio of the training set and testing set

	Ratio of training set and testing set		F1 score	Accuracy
	ratio	Testing set		
Case 1	60:40	792	0.989	0.989
Case 2	70:30	594	0.993	0.992
Case 3	75:25	495	0.988	0.988
Case 4	80:20	396	0.985	0.983
